# Distribution of aminopeptidase N coronavirus receptors in the respiratory and digestive tracts of domestic and wild artiodactyls and carnivores

**DOI:** 10.1099/jgv.0.002092

**Published:** 2025-04-04

**Authors:** Fabian Z.X. Lean, Giulia Gallo, Joseph Newman, Stuart Ackroyd, Simon Spiro, Ruth Cox, Ingebjørg Helena Nymo, Caroline Bröjer, Aleksija Neimanis, Alejandro Suárez-Bonnet, Simon L. Priestnall, Holly Everest, Sarah Keep, Dalan Bailey, Richard J. Delahay, Amanda H. Seekings, Lorraine M. McElhinney, Sharon M. Brookes, Alejandro Núñez

**Affiliations:** 1 Pathology and Animal Sciences Department, Animal and Plant Health Agency, Addlestone, UK; 2 Department of Pathobiology and Population Sciences, The Royal Veterinary College, North Mymms, UK; 3 The Pirbright Institute, Surrey, UK; 4 Zoological Society of London, London, UK; 5 National Wildlife Management Centre, Animal and Plant Health Agency, Sand Hutton, York, UK; 6 The Norwegian Veterinary Institute, Oslo, Norway; 7 Department of Pathology and Wildlife Diseases, Swedish Veterinary Agency, Uppsala, Sweden; 8 Virology Department, Animal and Plant Health Agency, Addlestone, UK

**Keywords:** aminopeptidase N, artiodactyla, carnivora, coronavirus

## Abstract

Aminopeptidase N (APN) is a transmembrane protein that mediates the attachment of the spike protein of several clinically important coronaviruses (CoVs) responsible for respiratory and intestinal diseases in animals and humans. To assess the potential for APN-mediated viral tropism, we characterized APN receptor distribution in the respiratory and intestinal tissues of various artiodactyls (cervids, bovids, camelids and suids) and carnivores (canids, felids, mustelids and phocids) using immunohistochemistry. In the lungs, APN expression was limited to artiodactyls, with strong expression in the bronchiolar epithelium and weaker expression in pneumocytes. Nasal turbinate and tracheal samples, where available, showed stronger APN expression in artiodactyls over carnivores. APN was consistently detected on the microvilli of enterocytes in the small intestine across multiple taxa, while the presence in the colon was more variable. Of the animals examined, pig and alpaca consistently expressed the most abundant APN in the upper and lower respiratory tract. *In silico* evaluation of APN orthologue sequences from humans, artiodactyls and carnivores identified distinct evolutionary relationships. Further *in silico* binding predictions for alpaca alphacoronavirus and human coronavirus 229E with cognate and heterologous alpaca and human APN revealed substantial overlapping binding footprints with high conservation of amino acid residues, suggesting an evolutionary divergence and subsequent adaptation of a 229E-like or ancestral virus within a non-human animal host. This combined anatomical and *in silico* approach enhances understanding of host susceptibility, tissue tropism and viral transmission mechanisms in APN-dependent CoVs and has the potential to inform future strategies for disease modelling, surveillance and control.

## Introduction

Aminopeptidase N (APN), also known as alanine aminopeptidase or CD13, is a transmembrane protein with an ectodomain that acts as a zinc-dependent, membrane-bound metalloprotease [1]. APN plays diverse biological functions, including immune regulation, inflammation, angiogenesis, cancer metastasis and serving as an entry receptor for coronaviruses (CoVs) of veterinary and medical importance [2,4]. While receptors such as angiotensin-converting enzyme 2 (ACE2; used by severe acute respiratory syndrome coronaviruses SARS-CoV and SARS-CoV-2) and dipeptidyl peptidase-4 (DPP4; used by Middle East Respiratory syndrome coronavirus MERS-CoV) have been extensively studied, the tissue distribution and conservation of APN, a key receptor mediating infections by alphacoronaviruses (*α*-CoVs) and deltacoronaviruses (*δ*-CoVs), remain less well-characterized [5,14].

APN-mediated CoVs include *α*-CoVs and *δ*-CoVs, which primarily affect non-human animals and are not associated with *β*- or *γ*-CoVs [15]. These viruses, such as feline coronavirus (FCoV), canine coronavirus (CCoV), transmissible gastroenteritis virus (TGEV), porcine epidemic diarrhoea virus (PEDV) and porcine deltacoronavirus (PDCoV), are predominantly enterotropic and are major causes of viral diarrhoeal diseases in young animals, which are either self-limiting or could result in significant morbidity and mortality [2]. However, some of these CoVs, such as porcine respiratory coronavirus (PRCV) and human coronavirus 229E (HCoV-229E), are respirotropic [2]. A shift from enterotropism to respirotropism has been demonstrated, as a spike (S) protein truncation in TGEV led to the emergence of PRCV [16]. Furthermore, pneumonia in humans has been linked to animal-derived, APN-dependent, recombinant CoVs, including CCoV, FCoV and PDCoV, highlighting their zoonotic potential [17,19].

The cross-species transmission potential of CoVs is well recognized. A notable example is the reverse zoonosis of SARS-CoV-2 to American mink and ferrets, resulting in substantially different clinical outcomes in these animals [20,21]. These outbreaks were initially unexpected due to predictions of limited ACE2 binding affinity to SARS-CoV-2 in these species [13,14, 22]. However, the distribution and abundance of virus receptors in tissues correlated with the localization of infection and lesions in the upper or lower respiratory tracts, findings subsequently confirmed through *in vivo* trials [7,23]. Similarly, for MERS-CoV (*β*-CoVs), differences in DPP4 receptor distribution across the respiratory tracts of humans, camels and sheep were linked to host susceptibility, variations in infection site and transmission potential [11,24]. These examples underscore the importance of evaluating receptor distribution and its biological correlates with site-specific infection.

The susceptibility of a host species to coronaviral infection is influenced by the binding affinity between the S protein and the receptor, as well as the availability of the receptor within the tissues [8,13, 14]. This study examines the distribution of APN in the respiratory and digestive tracts of artiodactyls, carnivores and chiropterans using immunohistochemistry. Additionally, *in silico* analysis was conducted to evaluate APN conservation and virus-binding domains in a subset of clinically important CoVs. By integrating these approaches, the study aims to elucidate receptor availability in tissues and assess host range, with a particular focus on the risks associated with cross-species transmission of APN-dependent CoVs.

## Methods

### Bioinformatic analysis of APN

APN peptide sequences from various species were retrieved from GenBank (see Table S1, available in the online Supplementary Material). The exact immunogen region of the human APN peptide within the C-terminal domain is not provided on the datasheet due to proprietary constraints. Therefore, amino acid homology was assessed using blast^®^, for a 100 amino acid immunogen within this domain. Evolutionary analyses of the full APN peptide sequence were conducted in mega11 [25]. Sequences were aligned using clustalw, and the best-fitting protein substitution model was determined. The optimal phylogenetic tree was then generated using the neighbour-joining method and 1,000 bootstrap replicates. For structural analysis, AlphaFold (AlphaFold Server) was used to predict the interaction between the ectodomain of alpaca APN (aAPN; GenBank: XP_006198515.1) and the receptor-binding domain (RBD) of alpaca coronavirus (ACoV) S protein (GenBank: AFI49431.1). Structural model representations were obtained using Pymol [26].

### Cell transfection

The expression of species-specific APN in cells was conducted as previously described [14]. A full-length APN gene from different mammalian species was synthesized and codon-optimized for expression in human cells, then subcloned into the pCAGGS vector with the addition of a HA tag sequence at the 3′ end of the ORF (Biobasic). A total of 500 ng of a subset of APN expression constructs (human *Homo sapiens*, sheep *Ovis aries*, Bactrian camel *Camelus bactrianus*, ferret *Mustela putorius furo*, dog *Canis lupus familiaris*, cat *Felis catus*, camel *Camelus dromedarius*, greater horseshoe bat *Rhinolophus ferrumequinum*, greater mouse-eared bat *Myotis myotis*) or an empty vector for mock transfection (pCAGGS) in OptiMEM (Gibco) was transfected into 6-well plates of baby hamster kidney (BHK-21) cells using TransIT-X2 transfection reagent (Mirus). Successful transfection and expression of all APN constructs in BHK-21 cells were previously validated by flow cytometry (data not shown). Briefly, 24 h post-transfection, the monolayers of *in vitro* transfected cells were fixed in 10% neutral-buffered formalin for 24 h, then manually re-suspended and centrifuged at 1,500 ***g***. The cell pellets were re-suspended in 2% agarose (Sigma) and allowed to set. Agarose-embedded cell pellets were then processed into formalin-fixed paraffin-embedded sections using routine histology methods [27].

### Animal tissues

Formalin-fixed paraffin-embedded tissues were obtained from histology archives held by the Animal and Plant Health Agency (UK), the Royal Veterinary College, the Zoological Society of London (UK), the Norwegian Veterinary Institute and the Swedish Veterinary Agency as per previous publications [5,6]. Tissue samples had been collected as part of routine veterinary investigations, health surveillance or from hunting and pest control operations, in compliance with local legislation. Of those species available, animals with known diseases or tissues in a poor state of preservation as determined by histology were excluded from the immunolabelling and analysis to avoid artefacts that could impact microscopic interpretation.

### Immunohistochemistry (IHC)

Paraffin tissue sections of 4 µm thickness were dewaxed and rehydrated through xylene and absolute alcohol, respectively, quenched for endogenous peroxidase with 3% hydrogen peroxide in methanol (VWR International) (15 min; room temperature, RT), before epitope unmasking using pH 9 Target Retrieval Solution (Dako) (10 min, 100 °C). Slides were blocked with normal goat (1/66 dilution) and swine (1/33 dilution) serum cocktail (Vector Laboratories) (20 min, RT) and assembled into cover plates to facilitate IHC using the Sequenza system (Epredia). Samples were then incubated with a primary anti-APN antibody (1/6,000 dilution; ab108382, Abcam) [28,29] or control rabbit IgG (1/80,000 dilution) (1 h, RT), followed by incubation with rabbit-specific Envision^™^ polymer (Dako), diluted with normal goat (1/66 dilution) and swine (1/33 dilution) serum cocktail, for 30 min at RT. Tissue sections were thereafter visualized using 3,3-diaminobenzidine (Sigma Aldrich) (10 min, RT). TBS with Tween [145 mM NaCl, 5 mM Tris(hydroxymethyl)methylamine, 0.1% w/v Tween^®^20, adjusted to pH 7.6 with 1 M HCl; Fisher Scientific; VWR International] was used for rinsing sections between incubations. Subsequently, sections were counterstained in Mayer’s haematoxylin (Pioneer Research Chemicals Ltd.), dehydrated in absolute alcohol and xylene and glass coverslips mounted using dibutyl phthalate xylene.

Immunohistochemically stained slides were examined by a blinded veterinary pathologist (F.Z.X.L.) using a conventional light microscope, with species randomly assigned for analysis. Staining abundance within respiratory epithelial cells or enterocytes was scored using the following scale: 0 (absent), 1 (<25% positive cells, minimal), 2 (25–50% positive cells, low), 3 (50–75% positive cells, moderate) and 4 (>75 % positive cells, abundant). For domestic pig and wild boar, where immunopositive mononuclear round cells were detected in the submucosa or propria regions, a minimum score of 1 for abundance was assigned. Staining intensity was scored as follows: 0 (absent), 1 (weak), 2 (moderate) and 3 (strong).

## Results

### Immunohistochemical validation of antibody against APN

IHC optimization was performed using a commercial rabbit monoclonal antibody raised against the C-terminal domain of the human APN peptide. The amino acid identity of the C-terminal domain of human APN with the species examined in this study was highly conserved, ranging between 80 and 90% identity (Table S1). IHC was optimized on porcine kidney tissue using either pH 6 or 9 antigen retrieval buffers. The pH 9 antigen retrieval produced a clearer signal-to-noise ratio, with the optimal antibody dilution achieved at 1:6,000 (Fig. S1a, b). Following this, IHC was evaluated for cross-reactivity with APN from other species using BHK-21 cells expressing species-specific, plasmid-driven APN proteins, including those from humans, Bactrian camels, sheep, ferrets, dogs, cats, greater mouse-eared bats and greater horseshoe bats (Table S1). Intense membranous and cytoplasmic immunolabelling was observed in cells expressing APN protein (Fig. S1c–j), with no immunolabelling detected in empty plasmid control cells (Fig. S1k).

IHC was further validated in kidney sections from each included species, which served as a positive internal control to confirm immunolabelling (Table S2). The kidney was selected as the target organ for IHC validation due to the abundance of APN expression [30,31]. The immunolabelling of APN in animal kidney sections was consistent across species, characterized by membranous labelling on the apical aspect of the proximal renal tubular epithelium (Fig. S1a). Non-specific immunolabelling was not detected when the anti-APN primary antibody was replaced with concentration-matched rabbit IgG (Fig. S1b). The use of kidney sections from each species as internal controls validated the immunohistochemical experiments.

### Immunohistochemical characterization of APN distribution in the respiratory tissues

**Fig. 1.**
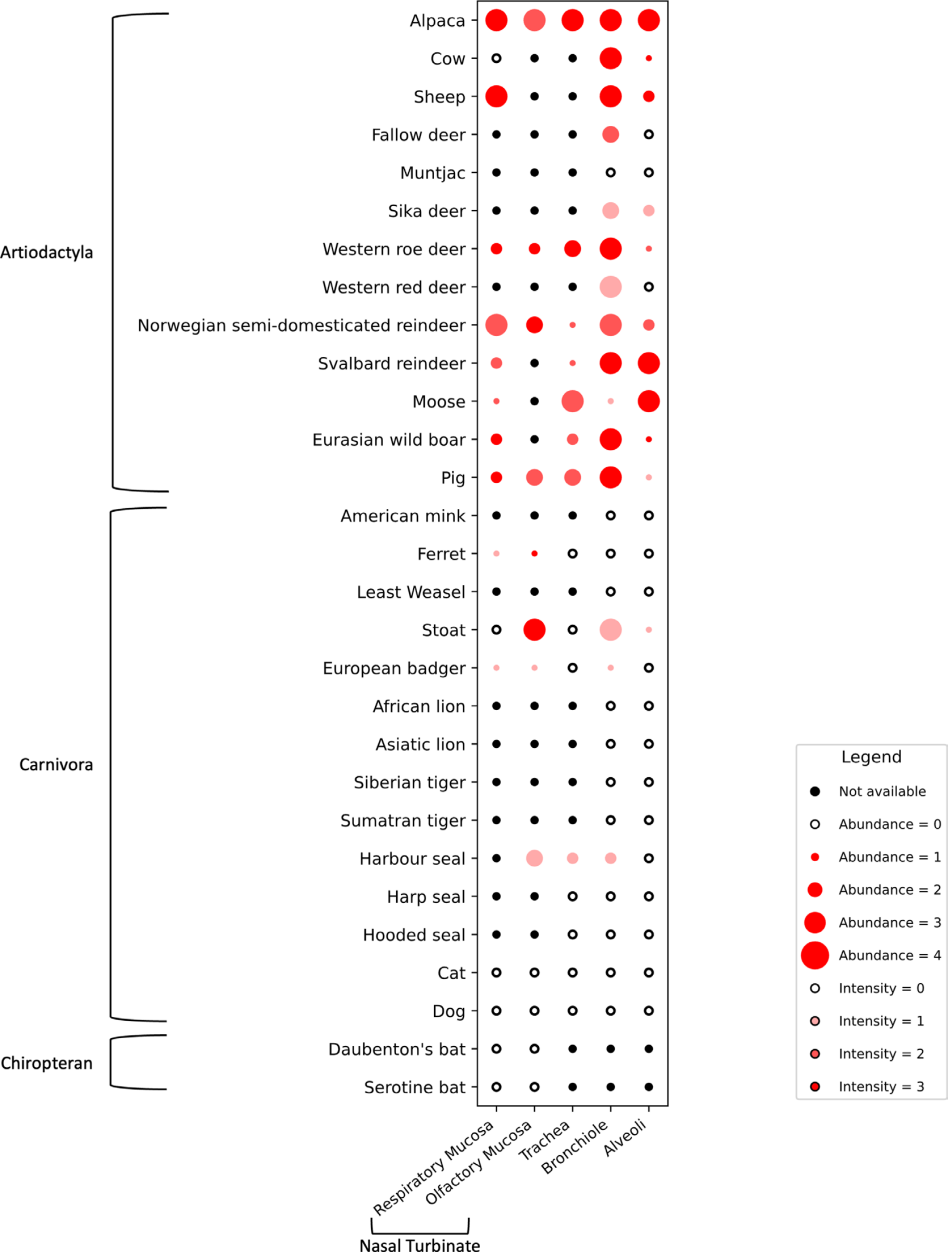
Immunohistochemical assessment of the abundance and intensity of APN immunolabelling in the respiratory tissues of artiodactyls, carnivores and chiropterans. The respiratory tissues examined included the nasal turbinate (respiratory and olfactory mucosa), trachea and lung (bronchiole and alveoli). Staining abundance within epithelial cells was scored as follows: 0 (absent); 1 (<25%, minimal); 2 (25–50%, low); 3 (50–75%, moderate); 4 (>75%, abundant). Staining intensity was scored as follows: 0 (absent); 1 (weak); 2 (moderate); 3 (strong). Species listed on the plot are organized based on clustering of family and genus and not indicative of the ranking of the results.

To investigate the spatial distribution and abundance of APN protein expression in the respiratory tract, lung tissues from a broad range of artiodactyls and carnivores, as well as nasal turbinate and tracheal tissues from a subset of species, were immunolabelled and assessed (Fig. 1 and Table S3).

APN immunolabelling in the lungs was widespread among artiodactyls. This included the bronchiolar epithelium of the alpaca (*Lama pacos*), pig (*Sus scrofa domesticus*), wild boar (*Sus scrofa*), cattle (*Bos taurus*), sheep, roe deer (*Capreolus capreolus*), red deer (*Cervus elaphus*), Svalbard reindeer (*Rangifer tarandus platyrhynchus*) and Norwegian semi-domesticated reindeer (*Rangifer tarandus tarandus*) (Figs1  2). Moose (*Alces alces*), on the other hand, had a low abundance of APN. The immunolabelling intensity was generally strong, although some species, such as the red deer, moose, fallow deer (*Dama dama*) and sika deer (*Cervus nippon*), exhibited moderate to low levels of APN on the bronchiolar epithelium. In the alveoli, the alpaca, Svalbard reindeer and moose expressed an abundant APN, with intense immunolabelling predominantly in the type II pneumocytes and often also in type I pneumocytes. Alveolar labelling was absent in the red, fallow and muntjac deer. In the pig, wild boar, cow, sheep and roe deer, APN abundance in the alveoli was generally minimal to low but with moderate to strong immunolabelling intensity in both type I and II pneumocytes.

**Fig. 2.**
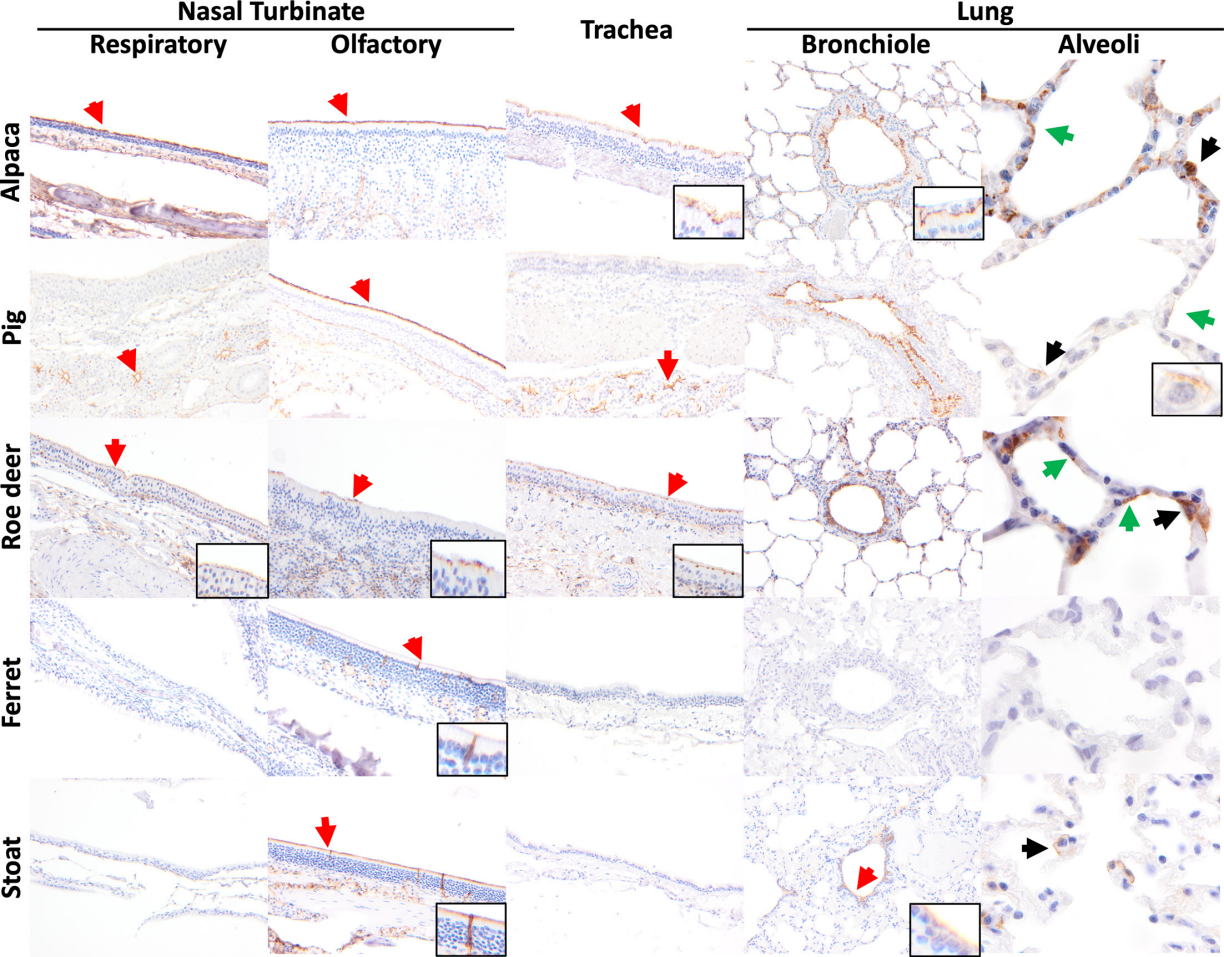
Immunohistochemical distribution of APN in the upper and lower respiratory tract of alpaca (*L. pacos*), pig (*S. scrofa domesticus*), roe deer (*C. capreolus*), ferret (*M. putorius furo*) and stoat (*M. erminea*). Positive APN immunolabelling is indicated by arrows. In the nasal turbinate, APN immunolabelling was detected in the respiratory epithelium (red arrow) of the alpaca and roe deer, with additional immunolabelling in the submucosal glands of the pig. In the olfactory mucosal epithelium, APN immunolabelling was detected in the alpaca, pig, roe deer and stoat, while sparse immunolabelling was noted in the olfactory neuronal cells of the ferret and stoat (insets). In the tracheal epithelium, APN immunolabelling was detected in the alpaca, pig and roe deer (red arrow). In the bronchiolar epithelium, abundant APN immunolabelling was detected in the alpaca, pig and roe deer, with a moderate amount of immunolabelling in the stoat. Within the alveoli, positive immunolabelling was detected in type I pneumocytes (green arrow) in the alpaca, pig and roe deer, and in type II pneumocytes (black arrow) in the alpaca, pig, roe deer and stoat. Images were taken at 200× (nasal turbinate, trachea, bronchiole) and 1,000× (alveoli and various insets to demonstrate immunolabelling) magnification.

**Fig. 3.**
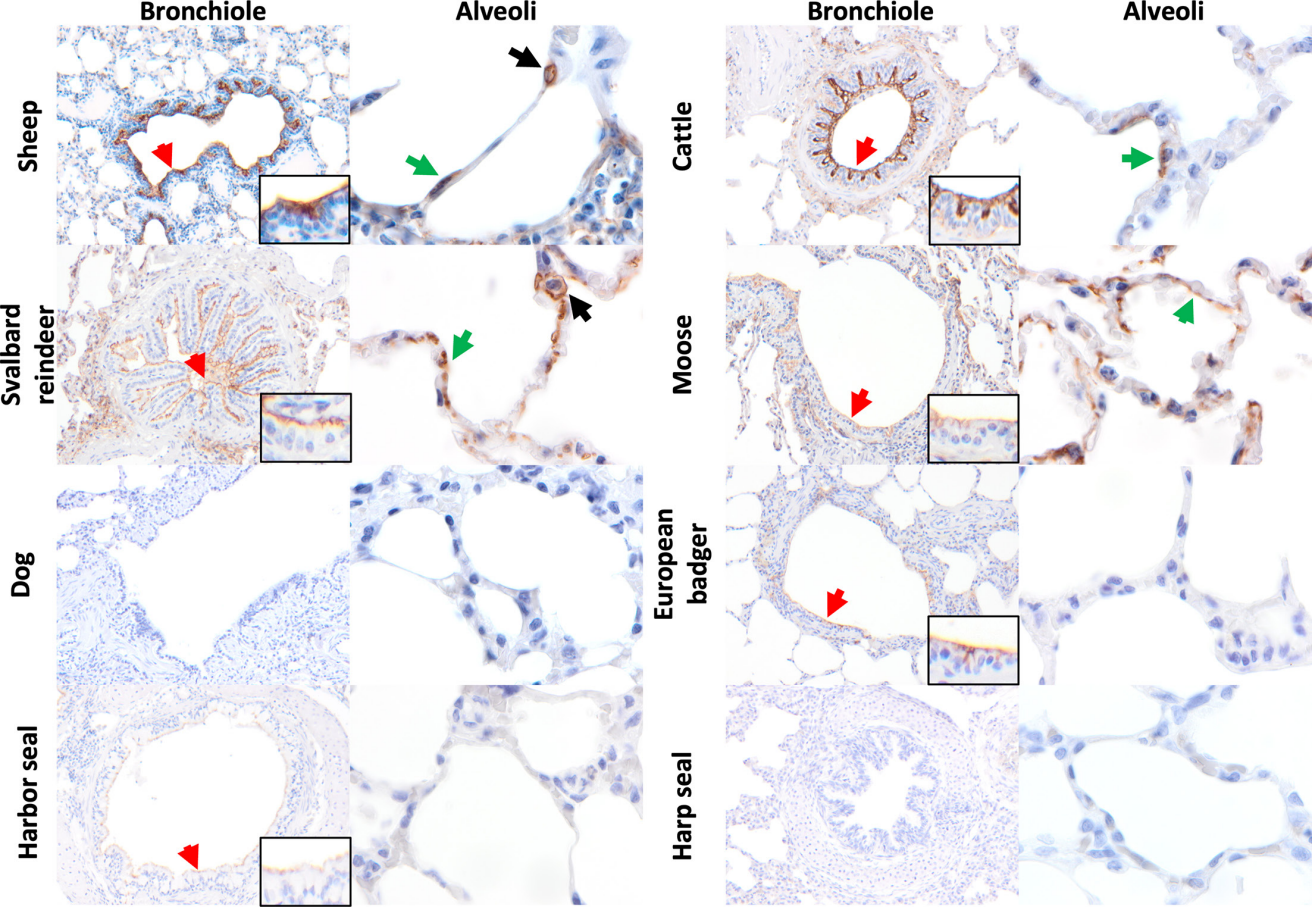
Immunohistochemical distribution of APN in the lung of a subset of artiodactyls, carnivores and phocids. Positive APN immunolabelling is indicated by arrows. In the bronchiole, APN immunolabelling was detected in the bronchiolar epithelium (red arrow) of sheep (*O. aries*), cattle (*B. taurus*), Svalbard reindeer (*R. t. platyrhynchus*), moose (*A. alces*), European badger (*M. meles*) and harbour seal (*P. vitulina*). In the alveoli, APN immunolabelling was observed in the type I pneumocytes (green arrow) of the sheep, cattle, reindeer and moose, as well as in the type II pneumocytes (black arrow) of the sheep and reindeer. No APN immunolabelling was detected in the lungs of the dog (*C. lupus familiaris*) and the harp seal (*P. groenlandicus*). Images were taken at 200× (bronchiole) and 1,000× (alveoli and insets to demonstrate immunolabelling) magnification.

In the lungs of carnivores, varying levels of APN expression were detected, though generally at low abundance or not detected. Moderate to low abundance of APN expression was observed in the stoat (*Mustela erminea*), specifically in the bronchiolar epithelium and type II pneumocytes in the alveoli (Fig. 2). In the European badger, the bronchiolar epithelium exhibited minimal abundance and weak immunolabelling for APN (Fig. 3). No APN immunolabelling was detected in the lungs of the American mink (*Neogale vison*), ferret, least weasel (*Mustela nivalis*), African lion (*Panthera leo*), Asiatic lion (*Panthera leo persica*), Siberian tiger (*Panthera tigris altaica*), Sumatran tiger (*Panthera tigris sondaica*), cat or dog.

Of the three phocid species examined, only the harbour seal (*Phoca vitulina*) exhibited a moderate abundance of APN immunolabelling, albeit with low intensity in the bronchiolar epithelium (Fig. 3). No immunolabelling was detected in the lungs of the harp seal (*Pagophilus groenlandicus*) or hooded seal (*Cystophora cristata*).

In species where nasal turbinate tissue was available, APN expression was detected at varying levels in the respiratory epithelium. Moderate to high abundance of APN was detected in the alpaca, sheep, and Norwegian semi-domesticated reindeer, and with low abundance in the pig, wild boar, moose, roe deer, Svalbard reindeer, ferret and European badger (*Meles meles*). Immunolabelling intensity was generally moderate to strong in these species, except for the European badger and ferret which were weak. In the pig and wild boar, in contrast to the sparse immunolabelling of the mucosal epithelium, widespread APN immunolabelling was detected in the submucosal glands (Fig. S2). Furthermore, rare immunopositive mononuclear round cells were detected within the submucosal of the respiratory nasal turbinate of both the pig and wild boar (Fig. S2), with cell morphology compatible with macrophages or dendritic cells. No immunolabelling was detected in the nasal turbinates of the dog, cat, serotine bat (*Eptesicus serotinus*) and Daubenton’s bat (*Myotis daubentonii*).

In the olfactory epithelium of the nasal turbinates, abundant APN immunolabelling was detected in the alpaca, pig, Norwegian semi-domesticated reindeer, stoat and harbour seal, while the roe deer, ferret and European badger showed minimal to low levels of labelling. Immunolabelling intensity ranged from weak to strong across these species, with the labelled cell types predominantly being olfactory mucosal epithelial cells. Infrequent immunolabelling of olfactory neuronal cells, characterized by their bipolar appearance, was detected in the ferret and stoat. In the ferret, only olfactory neuronal cells were immunolabelled, with no labelling of the mucosal epithelial cells. No APN immunolabelling was detected in the olfactory mucosa of the nasal turbinates in the dog or cat.

APN immunolabelling was detected in the tracheal epithelium of the alpaca, roe deer, and moose, with high abundance and moderate to strong intensity. In the wild boar, Svalbard and Norwegian semi-domesticated reindeer, and harbour seal, APN immunolabelling in the trachea was minimal to low. In the pig, there was an abundance of APN immunolabelling only in the submucosal glands but not within the tracheal mucosal epithelium. No APN immunolabelling was observed in the trachea of the ferret, stoat, European badger, harp and hooded seals, dog and cat.

### Immunohistochemical characterization of APN distribution in the intestine and colon

**Fig. 4.**
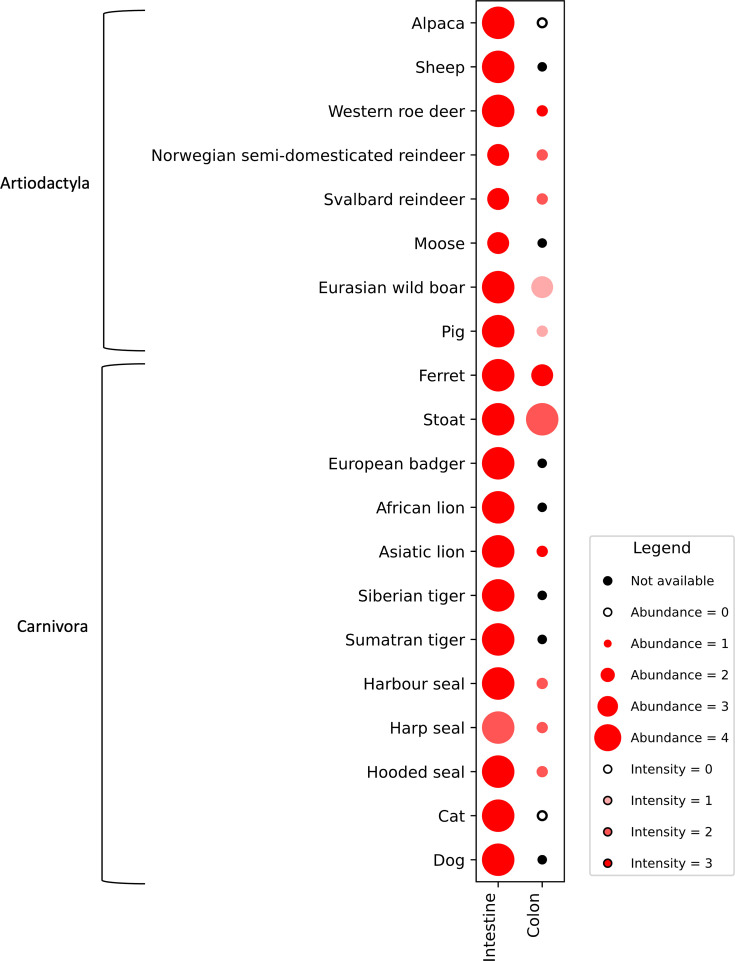
Immunohistochemical assessment of the abundance and intensity of APN immunolabelling in the intestine and colon of a subset of artiodactyls and carnivores. Staining abundance within enterocytes was scored as follows: 0 (absent); 1 (<25%, minimal); 2 (25–50%, low); 3 (50–75%, moderate); 4 (>75%, abundant). Staining intensity was scored as follows: 0 (absent); 1 (weak); 2 (moderate); 3 (strong). Species listed on the plot are organized based on clustering of family and genus and not indicative of the ranking of the results.

APN expression was detected in all intestinal tissue sections across a wide range of species, including alpaca, sheep, wild boar, pig, roe deer, Svalbard and Norwegian semi-domesticated reindeer, moose, dog, cat, European badger, stoat, ferret, Siberian tiger, Sumatran tiger, Asiatic lion, African lion, harbour seal, harp seal and hooded seal (Figs4  5). APN immunolabelling was abundant, with moderate to strong intensity, generally throughout the villus tip, while minimal immunolabelling was detected in the cryptal epithelium. Additionally, in the pig and wild boar, scattered APN-immunopositive round cells in the lamina propria were detected (Fig. S3). These round cells were larger than lymphocytes and thus suggestive of macrophages. Round cell immunolabelling was not detected in porcine tissue sections incubated with control rabbit IgG as the primary antibody.

**Fig. 5.**
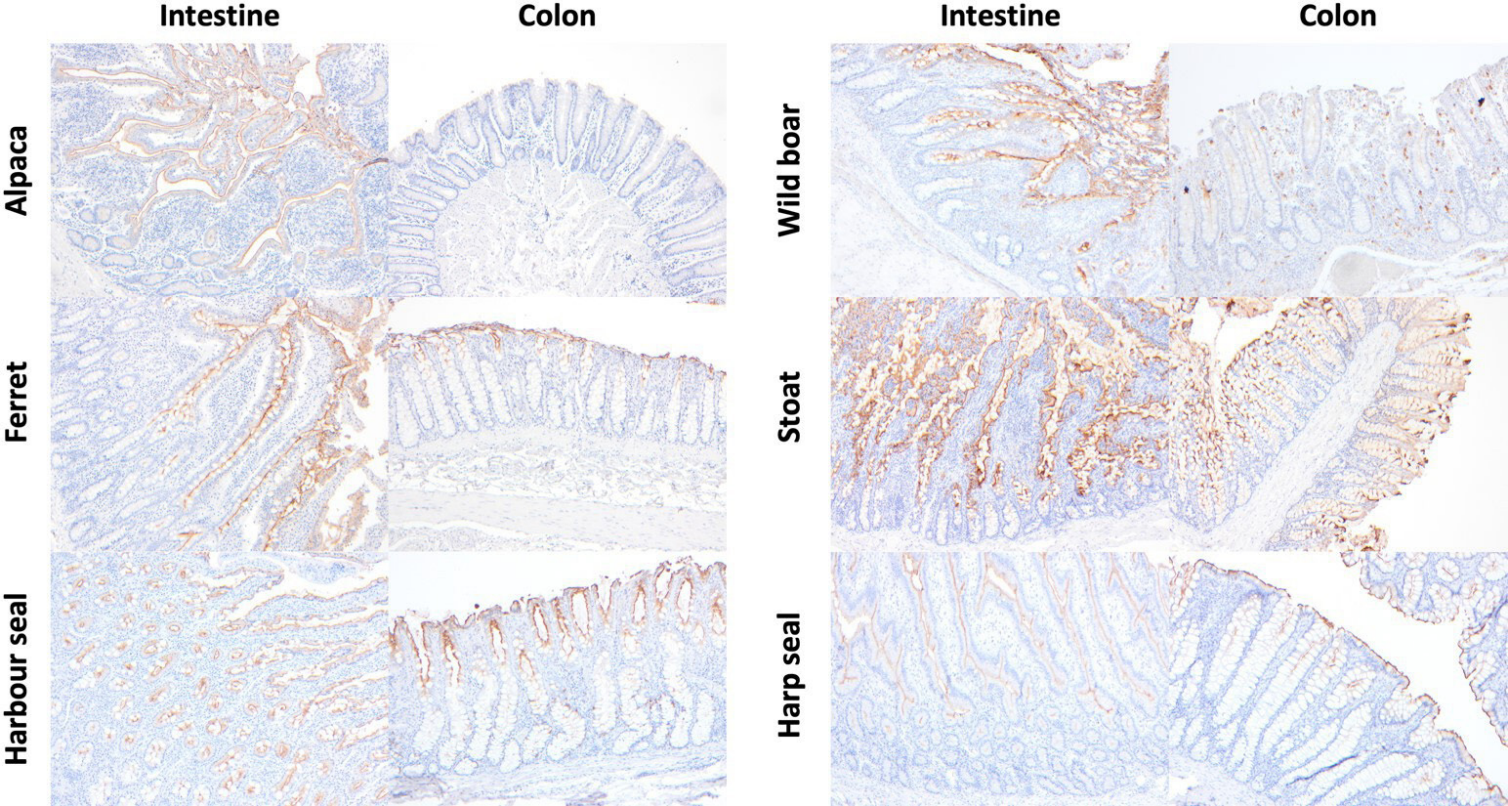
Immunohistochemical distribution of APN in the intestine and colon of a subset of artiodactyls, carnivores and phocids. In the intestine, APN immunolabelling was abundant along the villi and minimal in the cryptal epithelium in the alpaca, wild boar, ferret, stoat, harbour seal and harp seal. In the colon, APN was localized to the upper one-third and luminal aspects of the colonic glandular epithelial cells in the wild boar, harbour and harp seal, and approximately half of the luminal aspects in the stoat. No APN immunolabelling was detected in the colonic epithelium of the alpaca. Occasional macrophages within the lamina propria were immunolabelled in the wild boar (also see Fig. S3). Images were taken at 100× magnification.

APN expression in colonic sections varied across species, ranging from immunolabelling observed in the upper one-third or half of the colonic glandular epithelial cells to minimal presence in some instances. In the harbour seal, harp seal (Fig. 5), hooded seal, Svalbard and Norwegian semi-domesticated reindeer, roe deer and Asiatic lion, APN expression was restricted to the upper one-third of the colonic glandular epithelial cells. In the stoat and ferret, APN immunolabelling was observed in approximately the upper half of the colonic glandular epithelial cells. Minimal to no APN immunolabelling was detected in the colonic epithelium of pig, wild boar, alpaca and cat. Occasional proprial round cells were positively immunolabelled for APN in pig and wild boar (Fig. S3). Similar to the intestines, these cells resembled macrophages. There was no immunolabelling of the round cells within the colonic sections of pig and wild boar tissue when these were incubated with control rabbit IgG as the primary antibody.

### *In silico* analysis of APN peptide sequence and virus-binding domains

To examine the conservation of APNs in mammals, amino acid sequences from humans were aligned with those of various livestock (pig, cow, sheep) and animals known to be infected by CoVs that use APN as a receptor for entry (dog, cat, alpaca, dromedary camel) [2,32,34]. Additionally, sequences from animals implicated in other CoV infections were included, such as cervids (red deer, fallow deer), mustelids (ferret, American mink, European badger), large felids (tiger), pinnipeds [harbour seal, grey seal (*Halichoerus grypus*)] and chiropterans (Daubenton’s and greater horseshoe bats) [21,35,39].

**Fig. 6.**
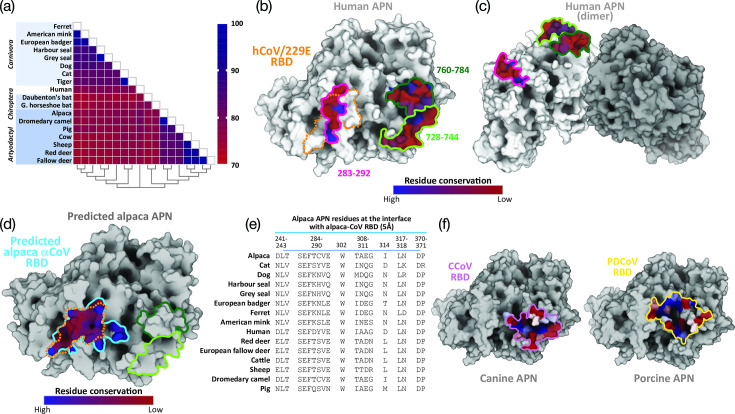
APN conservation among selected mammalian species and structural predictions of aAPN, highlighting the potential binding site of ACoV RBD. (a) Percentage peptide identity matrix, with species grouped by order on the *y*-axis, and the phylogenetic tree on the *x*-axis. (b) Previously identified CoV-binding domains on human APN, outlined in magenta (residues 283–292, VBM1), light green (residues 728–744, VBM2) and dark green (residues 760–784, VBM3) [1, 40], with residue conservation shown in a blue-to-red gradient (high to low conservation) based on the sequences listed in panel (a). AlphaFold predicted binding region of HCoV-229E RBD is demonstrated in dashed orange outline, showing extensive superposition on VBM1. (c) Side view of the dimeric form of human APN, illustrating the topographical proximity of VBM2 and VBM3 to the dimerization interface, while VBM1 is distal to the APN–APN interaction site. (d) AlphaFold prediction of the potential binding site for ACoV RBD on aAPN (solid blue outline) and superimposition of HCoV-229E RBD footprint (dashed orange outline), with residue conservation across animal species shown in a blue-to-red gradient. (e) Comparison of selected residues from different APNs at the predicted interaction interface between aAPN and ACoV RBD. (f) AlphaFold predictions of the potential binding sites of CCoV RBD on cAPN and PDCoV RBD on porcine APN, with residue conservation shown in the blue-to-red gradient as in panel (b, c, d).

Phylogenetic analysis of predicted protein sequences was performed using the neighbour-joining method (Fig. 6a). Two major clusters were identified, corresponding to the orders Carnivora (dogs, felids, mustelids and pinnipeds) and Artiodactyla (cervids, camelids, pigs, cows and sheep). The chiropteran formed a separate clade, which diverged separately from the Artiodactyla and is distant from Carnivora and humans. In the Artiodactyla cluster, a high level of amino acid identity was observed between alpacas and dromedary camels (98%), while identity among cattle, sheep and deer was ~92%. Mustelids also shared a high amino acid identity of up to 94%. In contrast, dogs and cats shared only 82% amino acid identity, and the two bat species examined (Daubenton’s and greater horseshoe bat) exhibited only 78% identity between the species.

In previous studies, the sites of human and porcine APN interacting with HCoV-229E and PRCV RBDs [1,40], respectively, have been resolved, highlighting three putative virus binding motifs (VBM). These comprise of VBM1 (Fig. 6b, outlined in magenta; residues 283–292; site recognized by HCoV-229E), VBM2 (Fig. 6b, outlined in light green; residues 728–744; site recognized by TGEV, FCoV and CCoV) and VBM3 (Fig. 6b, outlined in dark green; residues 760–784; site recognized by FCoV and CCoV). Based on the resolved 3D structure of human APN, VBM2 and VBM3 are localized near the dimerization interface, while VBM1 is distal (Fig. 6c).

Based on human APN (hAPN) VBM1, the core structure of VBM1 was relatively less conserved (red) compared to the extremities of the domain (blue) across the selected animal species (Fig. 6b, c). Clear clustering based on peptide sequence identity was observed for Artiodactyla, where camelids (alpaca and dromedary camel), bovids, ovine and cervids formed a distinct cluster with highly identical sequences (Table S4). In contrast, clustering within Carnivora was less pronounced, with a wider range of sequence identity (50–80%). For VBM2 and VBM3, the cores were more conserved than their peripheral regions, although overall conservation was generally limited (Fig. 6c). In VBM2, clustering by protein identity was evident only at the family level, with complete identity for cows and sheep, and for mustelids between 75 and 88% (Table S5). In VBM3, distinct clustering by protein identity was observed between Artiodactyla and Carnivora. Artiodactyls shared a narrower range of sequence identity (75–90%), while the Carnivora had a broader range of sequence identity (58–96%) (Table S6).

Because of the relevance of respiratory disease outbreaks among farmed alpacas associated with 229E-like ACoV [32,33], an Alphafold prediction of the ACoV RBD in complex with aAPN and HCoV-229E with hAPN was performed. The predicted binding footprint of the ACoV RBD on aAPN (interface predicted template modelling score=0.65, predicted template modelling score=0.81) overlapped with that of HCoV-229E, and the virus-binding interface was wider for ACoV compared to HCoV-229E (Fig. 6d, outlined in light blue and orange for alpaca and human VBM1, respectively). Based on this prediction, the residues of APN involved in the interaction with ACoV RBD were identified (Fig. 6e). Five out of twenty key residues differed between aAPN and human APN within the VBM1 interface: T287D, C288Y, T308I, E310A and I314D. It was shown that residue 288 of hAPN (aAPN 287) played a major role in determining the binding affinity of HCoV-229E [40]. This analysis suggested that the binding interfaces between ACoV-aAPN and HCoV-229E-hAPN were highly similar. The predicted interface residues for both HCoV-229E and ACoV were, however, poorly conserved across other species, which could potentially account for the narrow host range of HCoV-229E.

Given the zoonotic potential of CCoV and PDCoV [17,19], further AlphaFold predictions for CCoV with canine APN (cAPN) and PDCoV with porcine APN (pAPN) were performed (Fig. 6f). Both CCoV-cAPN and PDCoV-pAPN virus-binding footprints were distinct from those of ACoV-aAPN. The PDCoV-pAPN footprint closely resembled a previously reported model [41], while the CCoV-cAPN footprint in this study spanned residues 377–389 and 738–801, a broader region compared to the previously reported VBM2 and VBM3 (residues 728–784) [1]. Previous structural and mutagenesis studies have identified key residues on APN required for PDCoV infection, including hAPN 316 (pAPN 311), 379 (pAPN 374), 426 (pAPN 421) and 429 (pAPN 424) [41]. With the exception of residue 379 (pAPN 374), other three key residues were conserved among the human and animal species examined in this study (Table S7). However, additional residues identified in this study were varied. While the key binding residues for CCoV-APN have not been characterized through mutagenesis, the binding footprints of both CCoV-cAPN and PDCoV-pAPN were considerably variable and less conserved when compared across species, including humans (Fig. 6f and Tables S7 and S8).

## Discussion

With a surged in CoVs discovery following intensified research effort after the emergence of SARS-CoVs and MERS-CoV, other receptors utilized by CoVs for cell entry should be investigated to improve our understanding of host susceptibility. The anatomical distribution of APN receptors in tissues has only been evaluated in pigs due to the impact of *α*- and *δ*-CoVs infection in this production species [12,42]. The present study revealed a higher level of APN receptor expression in the respiratory tract of artiodactyls but limited expression in carnivores, which likely reflects the evolutionary divergence of these taxonomic groups. APN receptor expression in the respiratory tract of carnivores was observed only in a few species of mustelids and harbour seals. The expression of APN in the intestinal villi was consistently ubiquitous across different host species, while APN expression in the colonic glands was variable across species.

The upper and lower respiratory tracts of the alpaca have an abundance of APN expression. Previously, a novel alpaca *α*-CoV, closely related to HCoV-229E, was linked to acute pneumonia in alpacas [32,33]. The presence of APN in the bronchiolar epithelium and type I and II pneumocytes suggests that these areas may serve as primary infection sites, possibly accounting for the bronchointerstitial pneumonia and alveolar damage as reported in natural disease in the alpaca [32]. While the receptor usage of ACoV has not been previously evaluated, our *in silico* structural binding predictions revealed that the binding region on aAPN is highly similar to that of HCoV-229E and human APN, both of which have pneumonia potential in their respective hosts. While Koch’s postulates have not yet been fulfilled for ACoV in alpacas, these IHC findings, combined with the structural predictions, highlight the need for further investigation through viral IHC or *in situ* hybridization, as well as *in vivo* trials, to corroborate the role of APN in ACoV infections. Furthermore, while speculative, the evolutionary relationship between HCoV-229E and ACoV may parallel that of human coronavirus OC43 and bovine CoV, with divergence and adaptation of an ancestral virus in cloven-hoofed species [43,44].

Despite extensive research on HCoV-229E, the development of reliable animal models for studying experimental infections has been limited. Experimental infection of cats with 229E did not result in seroconversion [45]. Recent efforts have focused on using Ad5-hAPN-transduced or hAPN transgenic mice, although these models require immunocompromised backgrounds, such as interferon alpha/beta receptor (IFNAR) or signal transducer and activator of transcription (STAT)-deficient mice [46,47], and so may not accurately recapitulate the course of infection and host response. In contrast, alpacas have increasingly been used in *in vivo* and *ex vivo* studies of MERS-CoV, a virus of significant public health concern, to explore virus–host interactions, including immunological responses [48,50]. Given the presence of the APN receptor and the similar binding profiles between ACoV and HCoV-229E, alpacas offer a promising alternative for modelling natural CoV infections in immunocompetent hosts, whether as single or co-infections.

Of all species examined, pigs are the most well-characterized for APN-dependent virus infections, including the respirotropic PRCV. Within this context, bronchointerstitial pneumonia and PRCV tropism can be correlated to APN expression in the bronchial epithelium and type II pneumocytes [51]. However, while PRCV antigens were commonly detected in the respiratory and olfactory mucosal epithelium [51], APN was absent in the respiratory mucosa of the nasal turbinates and trachea, suggesting that PRCV may utilize alternative receptors in these regions, a hypothesis also proposed for PEDV, where APN knockout pigs did not confer protection against PEDV infection [52].

The respiratory tracts of mustelids have been examined with respect to their role in zoonotic and reverse zoonotic transmission of respiratory pathogens, such as COVID-19 in American mink and the use of ferrets in respiratory virus research [6,23]. In the present study, APN was detected in the nasal turbinates and lungs of stoats and European badgers, and in just the nasal turbinates of ferrets, but was absent from the lungs of American mink. Nasal turbinate tissue was not available for American mink, but given the consistent APN expression in other mustelids, it is plausible that they may also express APN in the upper respiratory tract. During the initial emergence of SARS-CoV-2, the mink and ferret were thought to be poorly susceptible to the virus due to weak ACE2 binding with S protein [13,14, 22], and yet *in vivo* trials and natural outbreaks in the mink contradicted this, likely in part due to the abundance of ACE2 that compensated for weak binding [6,7, 21, 23]. Although the APN sequence in mustelids differs from that in humans, the potential for APN-mediated viral infections in mustelids requires further investigation.

In this study, both dogs and cats show no detectable APN expression in the respiratory tract, with expression found only in the intestinal and colonic enterocytes. This could potentially correlate with the absence of respirotropic, APN-dependent CoVs in these species and the commonly understood enterotropism of CCoV and FCoV in these animals. Nevertheless, recent reports have demonstrated zoonotic transmission of recombinant CCoV and FCoV to humans, resulting in pneumonia in patients in Haiti and Malaysia, indicating the pneumotropic potential of these viruses [17,18]. Although systemic infection of CCoV in dogs has been reported in rare cases, respirotropism of CCoV and FCoV in their natural hosts has not been documented [53]. In this study, the binding footprints of CCoV-cAPN on hAPN were moderately conserved. Further assessment of the S proteins, their interactions and the target receptors, whether APN or alternative receptors, in the host of these virus isolates should be evaluated in the future. Nevertheless, based on current clinical data along with the findings of this study, the lack of APN expression in the respiratory tissues of dogs and cats, as determined by IHC, may confer resistance or a lack of susceptibility to viral respiratory infections, particularly endemic CCoV and FCoV, in these animals.

A large number of APN-dependent CoVs are predominantly enterotropic, and infections with these viruses, such as CCoVs and FCoVs, are typically subclinical or self-limiting [54,55]. However, they can cause significant mortality, particularly in piglets, where TGEV and PEDV lead to villus atrophy and diarrhoea [56,57]. Our study demonstrated widespread APN expression on the microvilli or apical aspects of enterocytes in the small intestine across various artiodactyl and carnivore species, which correlates with the tropism of the enteric CoVs. In contrast, APN immunolabelling in the colon was confined to the upper one-third to half of the luminal aspects of colonic glandular epithelial cells, or in some species, minimal to no APN labelling. Although the small intestine is the primary site of infection of a large number of CoVs, the colon could also serve as a replication site, typically without inducing significant lesions. Whilst widespread APN expression in the intestine is consistent across species and resembles the expression patterns of ACE2 and DPP4 [6,10], variations in APN expression in terms of abundance and cell type, particularly in the colonic tracts of pigs, could have implications for CoV pathogenesis.

In addition to epithelial labelling in the respiratory and digestive tracts, rare APN-positive mononuclear round cells were detected in the nasal turbinate submucosa and the intestinal and colonic lamina propria of pig and wild boar. While the exact identity of these cells was not confirmed, their size and morphology suggest they are likely macrophages or dendritic cells, rather than lymphocytes. A previous *in vivo* study of pigs intranasally inoculated with PEDV demonstrated virus-positive dendritic cells in the nasal turbinate submucosa during the early stages of infection, potentially contributing to subsequent viraemia and virus dissemination to the intestine [58]. Other studies have also reported PEDV antigens in proprial macrophages and demonstrated that PEDV infection of porcine macrophages can inhibit interferon responses [59,60]. In the current study, non-specific labelling in porcine tissues was considered, but it would involve lymphocytes due to Fc binding; however, control sections with rabbit IgG instead of the primary anti-APN antibody showed no labelling in any round cell population, supporting the specificity of the observed APN expression.

Some newly discovered CoVs, including those from the bat, are suggested to pose a potential risk of cross-species transmission, particularly among species within the order Artiodactyla [61], as was the case for MERS-CoV [62]. In the present study, IHC findings revealed that many artiodactyl species, including alpacas, express high levels of APN in their respiratory tracts and intestinal and colonic tissues. Interest in alpaca farming and their popularity as pets or show animals have grown significantly over the past decade [63]. While alpacas are generally raised outdoors at relatively low densities compared to some captive farmed animals, such as American mink or raccoon dogs, species whose confined conditions promote the transmission of respiratory virus infections [64,66], alpacas may also be kept in multi-species grazing systems or petting zoos, increasing their exposure to other cloven-hoofed animals and humans. Given the substantial expression of APN in livestock species, particularly alpacas, the potential risk of cross-species transmission of CoVs should not be overlooked.

This study is limited by the lack of nasal turbinate and tracheal samples from a broad range of animal species, as these tissues are rarely collected during routine necropsies. Future studies should prioritize their inclusion to provide a more comprehensive analysis. Expanding tissue collection to include other camelids, common laboratory animals and non-human primates is also necessary to assess their susceptibility to CoV infections to address natural disease infection and potential alternative animal models to advance CoV modelling. Given that HCoV-229E-like *α*-CoV have been detected in dromedary camels [34], mapping the APN distribution in tissues of Old World camelids and other New World camelids along with virus-binding assessment should be a priority. Furthermore, IHC has inherent sensitivity limitations, particularly with protein sequences that diverge from the immunogen. Thus, further studies using transcript expression analysis and *in situ* hybridization are essential to verify the IHC findings.

In conclusion, while the number of APN-mediated CoVs of current veterinary interest remains limited and is primarily focused on pigs, the increasing detection of novel CoVs and changes in animal production practices, such as the rising popularity of alpacas, could create opportunities that drive virus evolution. While understanding and predicting potential APN usage is multifactorial, integrating spatial distribution of APN at the tissue level, complementing with further assessment of binding sites for virus–host receptor interactions, will provide a more biologically meaningful evaluation of at-risk species or potential host species. Further investigations, including *in vitro* virus-receptor binding assessments and *in vivo* studies, are necessary to understand the implications of APN expression on host susceptibility and cross-species transmission dynamics, which may ultimately guide risk assessments for APN receptor-mediated viral infections in animals, including humans.

## supplementary material

10.1099/jgv.0.002092 Uncited Supplementary Material 1.

## Abbreviations

aAPN: alpaca APN
ACE2: angiotensin-converting enzyme 2
ACoV: alpaca coronavirus
APN: aminopeptidase N
BHK-21: baby hamster kidney
cAPN: canine APN
CCoV: canine coronavirus
CoVs: coronaviruses
DPP4: dipeptidyl peptidase-4
FCoV: feline coronavirus
hAPN: human APN
HCoV-229E: human coronavirus 229E
IHC: immunohistochemistry
MERS-CoV: Middle East Respiratory syndrome coronavirus
pAPN: porcine APN
PDCoV: porcine deltacoronavirus
PEDV: porcine epidemic diarrhoea virus
PRCV: porcine respiratory coronavirus
RBD: receptor-binding domain
RT: room temperature
S: spike
TGEV: transmissible gastroenteritis virus
VBM: virus-binding motifs
*α*-CoVs: alphacoronaviruses
*δ*-CoVs: deltacoronaviruses
