# Supplementary material for: Distribution of aminopeptidase N coronavirus receptors in the respiratory and digestive tracts of domestic and wild artiodactyls and carnivores
Source: J Gen Virol. 2025 Apr 4;106(4):002092. doi: 10.1099/jgv.0.002092 (PMC11971486; doi:10.1099/jgv.0.002092)
Supplement: Uncited Supplementary Material 1. [file jgv-106-02092-s001.pdf]

Table S1. Aminopeptidase N (APN) amino acid sequences used for homology alignment against APN immunogen sequence used for raising the rabbit monoclonal antibody or advance *in silico* analyses

**Common Name**	**Scientific Name**	**Peptide Sequence Identification**	**Identity (%)** [a]
Human [b]	*Homo sapiens*	NP_001368852.1	N.A [c]
Cat [b]	*Felis catus*	NP_001009252.2	88.73%
Dog [b]	*Canis lupus familiaris*	NP_001139506.1	88.89%
Ferret [b]	*Mustela putorius furo*	XP_012917463.1	85.92%
American mink [b]	*Neovison vison*	XP_044086346.1	87.50%
European badger [b]	*Meles meles*	XP_045864142.1	84.72%
Tiger	*Panthera tigris*	XP_042843592.1	90.14%
Lion	*Panthera leo*	XP_042796394.1	90.14%
Alpaca [b]	*Vicugna pacos*	XP_006198515.1	90.14%
Bactrian camel	*Camelus bactrianus*	XP_010953645.2	90.14%
Dromedary camel [b]	*Camelus dromedarius*	XP_031296423.1	90.14%
Cattle [b]	*Bos Taurus*	XP_024837385.1	81.69%
Sheep [b]	*Ovis aries*	XP_014957374.3	85.92%
Pig [b]	*Sus scrofa domestica*	NP_999442.1	85.92%
Fallow deer	*Dama dama*	XP_061014681.1	81.69%
Red deer [b]	*Cervus elaphus*	XP_043778071.1	81.69%
Harbor seal [b]	*Phoca vitulina*	XP_032247934.1	84.51%
Grey seal [b]	*Halichoerus grypus*	XP_035925810.1	84.51%
Greater mouse-eared bat	*Myotis myotis*	XP_036211683.1	85.92%
Greater horseshoe bat [b]	*Rhinolophus ferrumequinum*	XP_032956109.1	86.11%
Daubenton's bat [b]	*Myotis daubentonii*	XP_059534043.1	86.11%

[a] amino acid homology was assessed against the 100-amino-acid immunogen within the C-terminal domain of the human APN peptide, which was used to generate the antibody. The exact immunogen region of the rabbit anti-human APN antibody (ab108382, Abcam) remains proprietary information at publication.

[b] sequences used for *in silico* analysis of APN peptide sequence and virus binding domains

[c] not applicable

Table S2. Immunohistochemical cross-reactivity of human Aminopeptidase N (APN) polyclonal antibody with APN proteins from other mammals in formalin-fixed, paraffin-embedded tissue sections

**Common name**	**Scientific Name**	**BHK-21 Transfected Cells**	**Kidney Sections**
Human	*Homo sapiens*	+	N.P.
Cat	*Felis catus*	+	+
Dog	*Canis lupus familiaris*	+	+
Ferret	*Mustela putorius furo*	+	+
American mink	*Neovison vison*	N.P.	+
European Badger	*Meles meles*	N.P.	+
Stoat	*Mustela erminea*	N.P.	+
Siberian tiger	*Panthera tigris altaica*	N.P.	+
Sumatran tiger	*Panthera tigris sondaica*	N.P.	+
African lion	*Panthera leo*	N.P.	+
Asiatic lion	*Panthera leo leo*	N.P.	+
Alpaca	*Lama pacos*	N.P.	+
Bactrian camel	*Camelus bactrianus*	+	N.P.
Domestic cattle	*Bos taurus*	N.P.	+
Sheep	*Ovis aries*	+	+
Pig	*Sus scrofa domestica*	N.P.	+
Wild boar	*Sus scrofa*	N.P.	+
Western roe deer	*Capreolus capreolus*	N.P.	+
Moose	*Alces alces*	N.P.	+
Svalbard wild reindeer	*Rangifer tarandus platyrhynchus*	N.P.	+
Norwegian semi-domesticated reindeer	*Rangifer tarandus tarandus*	N.P.	+
Harbor seal	*Phoca vitulina*	N.P.	+
Harp seal	*Pagophilus groenlandicus*	N.P.	+
Hooded seal	*Cystophora cristata*	N.P.	+
Greater horseshoe bat	*Rhinolophus ferrumequinum*	+	N.P.
Greater mouse-eared bat	*Myotis myotis*	+	N.P.

Definition: + = immunopositive; N.P. = not performed

Table S3. Animal species and sample size of tissues examined by immunohistochemistry

Species	Respiratory Tissue	Small Intestine	Colon
Alpaca	3	3	3
Eurasian wild boar	3	3	3
Pig	3	3	3
Cow	2	N.A.	N.A.
Sheep	1	1	N.A.
Western roe deer	3	3	1
Western red deer	3	N.A.	N.A.
Fallow deer	3	N.A.	N.A.
Muntjac	2	N.A.	N.A.
Sika deer	3	N.A.	N.A.
Svalbard reindeer	4	2	1
Norwegian semi-domesticated reindeer	2	2	2
Moose	4	1	N.A.
American mink	3	N.A.	N.A.
Ferret	3	3	3
Least Weasel	3	N.A.	N.A.
Stoat	3	3	3
European badger	3	2	N.A.
African lion	2	2	N.A.
Asiatic lion	1	1	1
Siberian tiger	2	2	N.A.
Sumatran tiger	1	1	N.A.
Harbour seal	2	2	2
Harp seal	4	4	4
Hooded seal	5	2	4
Cat	3	3	2
Dog	3	3	N.A.
Daubenton's bat	1	N.A.	N.A.
Serotine bat	3	N.A.	N.A.

Definition: N.A. = not available

Table S4. Amino acid identity matrix for Virus Binding Motif (VBM) 1 (residues 283-292)

Percent Homology	Human	Alpaca	Dromedary camel	Pig	Cow	Sheep	Red deer	Fallow deer	Dog	Cat	Tiger	Ferret	American mink	European badger	Harbour seal	Grey seal	Daubenton's bat	Greater horseshoe bat
**Human**		70	70	60	70	70	70	70	60	80	80	70	60	60	60	60	70	70
**Alpaca**	70		100	60	80	80	80	80	60	70	70	80	60	60	60	60	70	80
**Dromedary camel**	70	100		60	80	80	80	80	60	70	70	80	60	60	60	60	70	80
**Pig**	60	60	60		70	70	70	70	70	60	60	70	60	50	60	60	70	70
**Cow**	70	80	80	70		100	100	100	60	70	70	90	70	60	60	60	70	90
**Sheep**	70	80	80	70	100		100	100	60	70	70	90	70	60	60	60	70	90
**Red deer**	70	80	80	70	100	100		100	60	70	70	90	70	60	60	60	70	90
**Fallow deer**	70	80	80	70	100	100	100		60	70	70	90	70	60	60	60	70	90
**Dog**	60	60	60	70	60	60	60	60		60	60	60	60	70	80	70	60	60
**Cat**	80	70	70	60	70	70	70	70	60		100	70	60	60	70	70	70	70
**Tiger**	80	70	70	60	70	70	70	70	60	100		70	60	60	70	70	70	70
**Ferret**	70	80	80	70	90	90	90	90	60	70	70		70	60	60	60	70	100
**American mink**	60	60	60	60	70	70	70	70	60	60	60	70		90	60	50	50	70
**European badger**	60	60	60	50	60	60	60	60	70	60	60	60	90		60	50	50	60
**Harbour seal**	60	60	60	60	60	60	60	60	80	70	70	60	60	60		90	60	60
**Grey seal**	60	60	60	60	60	60	60	60	70	70	70	60	50	50	90		60	60
**Daubenton's bat**	70	70	70	70	70	70	70	70	60	70	70	70	50	50	60	60		70
**Greater horseshoe bat**	70	80	80	70	90	90	90	90	60	70	70	100	70	60	60	60	70	

Table S5. Amino acid identity matrix for Virus Binding Motif (VBM) 2 (residues 728-744)

Percent Homology	Human	Alpaca	Dromedary camel	Pig	Cow	Sheep	Red deer	Fallow deer	Dog	Cat	Tiger	Ferret	American mink	European badger	Harbour seal	Grey seal	Daubenton's bat	Greater horseshoe bat
Human		59	59	59	59	59	59	59	53	53	53	47	41	53	47	47	53	59
Alpaca	59		100	88	65	65	59	59	71	71	71	59	47	59	65	65	71	59
Dromedary camel	59	100		88	65	65	59	59	71	71	71	59	47	59	65	65	71	59
Pig	59	88	88		71	71	65	59	65	71	76	71	59	53	65	65	76	59
Cow	59	65	65	71		100	88	59	53	59	65	65	53	59	59	59	53	47
Sheep	59	65	65	71	100		88	59	53	59	65	65	53	59	59	59	53	47
Red deer	59	59	59	65	88	88		59	53	53	59	65	53	53	59	59	53	47
Fallow deer	59	59	59	59	88	88	88		53	53	53	47	41	53	47	47	53	59
Dog	53	71	71	65	53	53	53	53		82	76	71	59	71	59	59	65	59
Cat	53	71	71	71	59	59	53	53	82		88	65	53	59	65	65	71	65
Tiger	53	71	71	76	65	65	59	53	76	88		76	65	65	65	65	65	53
Ferret	47	59	59	71	65	65	65	47	71	65	76		88	82	71	71	71	47
American mink	41	47	47	59	53	53	53	41	59	53	65	88		76	59	59	59	41
European badger	53	59	59	53	59	59	53	53	71	59	65	82	76		65	65	59	47
Harbour seal	47	65	65	65	59	59	59	47	59	65	65	71	59	65		100	71	59
Grey seal	47	65	65	65	59	59	59	47	59	65	65	71	59	65	100		71	59
Daubenton's bat	53	71	71	76	53	53	53	53	65	71	65	71	59	59	71	71		65
Greater horseshoe bat	59	59	59	59	47	47	47	59	59	65	53	47	41	47	59	59	65	

Table S6. Amino acid identity matrix for Virus Binding Motif (VBM) 3 (residues 760-784)

Percent Homology	Human	Alpaca	Dromedary camel	Pig	Cow	Sheep	Red deer	Fallow deer	Dog	Cat	Tiger	Ferret	American mink	European badger	Harbour seal	Grey seal	Daubenton's bat	Greater horseshoe bat
**Human**		54.17	54.17	58.33	66.67	62.5	66.67	66.67	54.17	62.5	54.17	58.33	58.33	58.33	58.33	58.33	58.33	58.33
**Alpaca**	54.17		100	75	83.33	83.33	83.33	83.33	50	58.33	62.5	50	54.17	54.17	50	50	54.17	41.67
**Dromedary camel**	54.17	100		75	83.33	83.33	83.33	83.33	50	58.33	62.5	50	54.17	54.17	50	50	54.17	41.67
**Pig**	58.33	75	75		79.17	79.17	75	75	45.83	62.5	58.33	41.67	45.83	45.83	45.83	45.83	58.33	45.83
**Cow**	66.67	83.33	83.33	79.17		87.5	91.67	91.67	54.17	66.67	66.67	54.17	58.33	58.33	58.33	58.33	54.17	50
**Sheep**	62.5	83.33	83.33	79.17	87.5		91.67	91.67	50	62.5	62.5	50	54.17	54.17	54.17	54.17	54.17	50
**Red deer**	66.67	83.33	83.33	75	91.67	91.67		100	50	62.5	62.5	50	54.17	54.17	54.17	54.17	50	45.83
**Fallow deer**	66.67	83.33	83.33	75	91.67	91.67	100		50	62.5	62.5	50	54.17	54.17	54.17	54.17	50	45.83
**Dog**	54.17	50	50	45.83	54.17	50	50	50		66.67	75	87.5	91.67	91.67	83.33	83.33	54.17	54.17
**Cat**	62.5	58.33	58.33	62.5	66.67	62.5	62.5	62.5	66.67		91.67	66.67	70.83	70.83	58.33	58.33	58.33	54.17
**Tiger**	54.17	62.5	62.5	58.33	66.67	62.5	62.5	62.5	75	91.67		75	79.17	79.17	62.5	62.5	54.17	50
**Ferret**	58.33	50	50	41.67	54.17	50	50	50	87.5	66.67	75		95.83	95.83	79.17	79.17	54.17	54.17
**American mink**	58.33	54.17	54.17	45.83	58.33	54.17	54.17	54.17	91.67	70.83	79.17	95.83		100	83.33	83.33	54.17	54.17
**European badger**	58.33	54.17	54.17	45.83	58.33	54.17	54.17	54.17	91.67	70.83	79.17	95.83	100		83.33	83.33	54.17	54.17
**Harbour seal**	58.33	50	50	45.83	58.33	54.17	54.17	54.17	83.33	58.33	62.5	79.17	83.33	83.33		100	58.33	54.17
**Grey seal**	58.33	50	50	45.83	58.33	54.17	54.17	54.17	83.33	58.33	62.5	79.17	83.33	83.33	100		58.33	54.17
**Daubenton's bat**	58.33	54.17	54.17	58.33	54.17	54.17	50	50	54.17	58.33	54.17	54.17	54.17	54.17	58.33	58.33		66.67
**Greater horseshoe bat**	58.33	41.67	41.67	45.83	50	50	45.83	45.83	54.17	54.17	50	54.17	54.17	54.17	54.17	54.17	66.67	

Table S7. Contact residues of PDCoV within 5 Å of porcine APN and homology comparison with APN from other species

**Amino Acid Positions**	**310-311**	**314**	**364**	**366-367**	**374**	**421**	**423-424**	**732**	**735**	**738**	**742-744**	**746-747**	**783-787**	**790**
**Pig**	MY	N	F	PQ	K	E	TW	T	K	T	ENL	DQ	NPIHP	R
Alpaca	IY	N	F	PL	K	E	TW	S	K	T	QNL	DQ	NPIHP	R
Dog	NY	R	Y	PQ	K	E	TW	K	Q	T	QTL	EQ	NPIYP	R
Harbor Seal	NY	N	F	PQ	Q	E	TW	G	Y	T	QTL	EQ	NPIHP	R
Grey Seal	NY	N	F	PQ	Q	E	TW	G	Y	T	QTL	EQ	NPIHP	R
European badger	TY	N	Y	PQ	K	E	TW	N	Q	M	DTL	EQ	NPIYP	R
Ferret	NY	D	Y	PL	R	E	SW	T	Q	T	DTL	EQ	NSIYP	R
American mink	NY	N	Y	PL	R	E	TW	T	Q	K	DTL	EQ	NPIYP	R
Human	DY	N	F	PL	K	E	TW	N	N	R	ENL	DQ	NPIHP	R
Red deer	LY	N	Y	PQ	K	E	TW	Q	N	T	ENL	DQ	NPIDP	R
Fallow deer	LY	N	Y	PQ	K	E	TW	Q	K	T	ENL	DQ	NPIDP	R
Cattle	LY	N	Y	PQ	K	E	TW	N	K	T	ENL	DQ	NPIDP	R
Sheep	LY	N	Y	PQ	K	E	TW	N	K	T	ENL	DQ	NPINP	R
Dromedarius	IY	N	F	PL	K	E	NW	S	K	T	QNL	DQ	NPIHP	R

Table S8. Contact residues of CCoV within 5 Å of canine APN and homology comparison with APN from other species

Amino Acid Positions	377-379	738	742	746-750	775	778-779	782-783	786	789	793-798	801
Dog	PQS	F	E	QNWTD	K	DL	TL	E	K	NNPIYP	R
Alpaca	PLS	F	E	KNWTE	K	EL	TL	N	K	NNPIHP	R
Harbor Seal	PQS	F	E	YNWTV	K	EL	TL	K	R	NNPIHP	R
Grey Seal	PQS	F	E	YNWTV	K	EL	TL	K	R	NNPIHP	R
European badger	PQS	F	E	QNWMK	K	EL	TL	E	K	NNPIYP	R
Ferret	PLS	F	E	QNWTK	K	EL	AL	E	K	NNSIYP	R
American mink	PLS	F	E	QGWKK	K	EL	TL	E	K	NNPIYP	R
Human	PLS	F	R	NNWRE	E	EM	GL	Q	E	NNPIHP	R
Red deer	PQS	F	E	NNWTE	K	EL	TL	Q	N	VNPIDP	R
Fallow deer	PQS	F	E	KNWTE	K	EL	TL	Q	N	VNPIDP	R
Cattle	PQS	F	E	KNWTE	K	EL	TL	Q	N	VNPIDP	R
Sheep	PQS	F	E	KNWTE	K	EL	TL	Q	S	VNPINP	R
Dromedarius	PLS	F	E	KNWTE	K	EL	TL	N	K	NNPIHP	R
Pig	PQS	F	E	KNWTE	Q	NL	TL	Q	S	NNPIHP	R

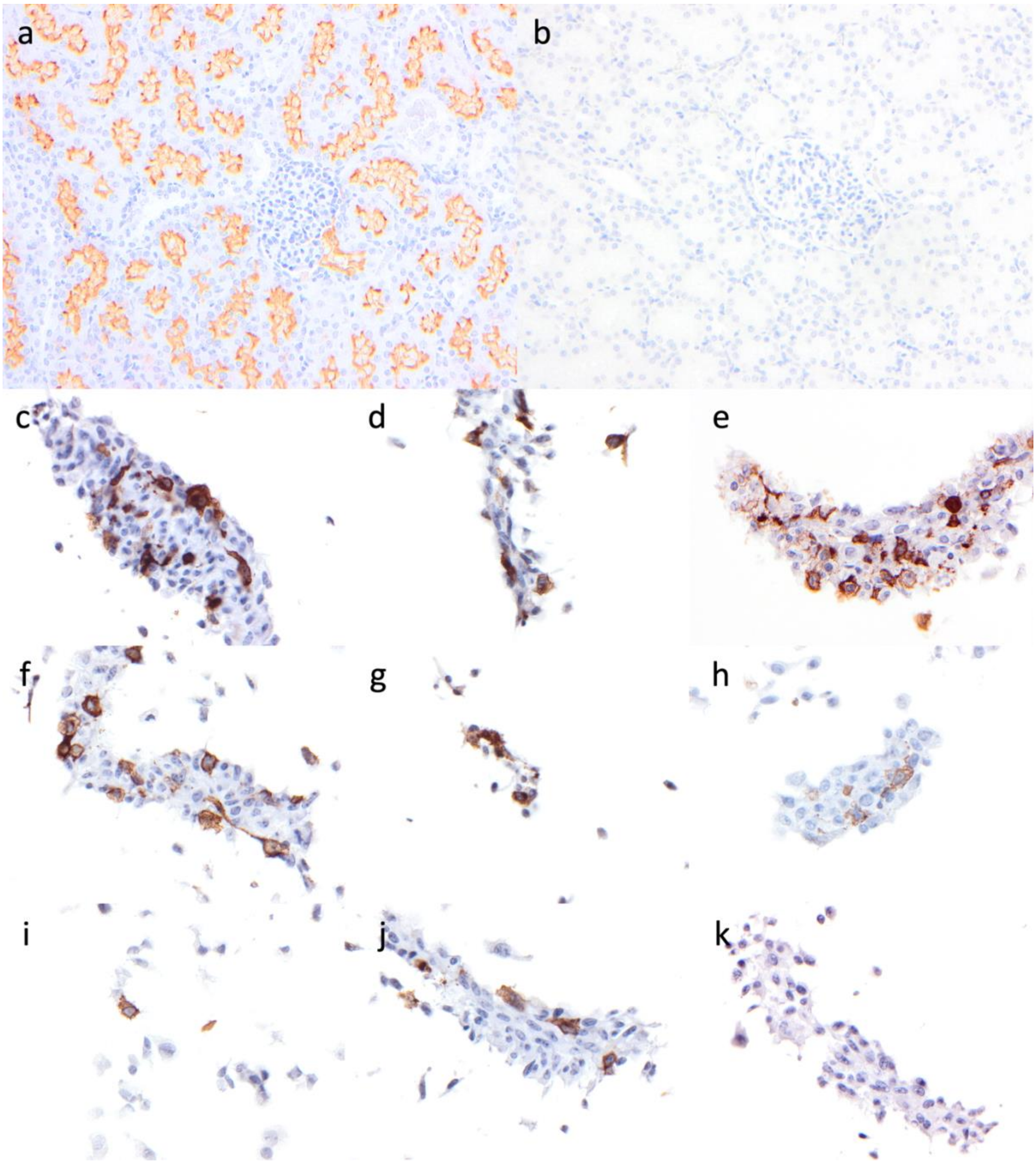

Figure S1. Confirmation of Aminopeptidase N (APN) immunolabeling specificity using kidney sections and APN-transfected BHK-21 cells. Positive membranous immunolabeling was detected on the apical aspect of renal tubular epithelium in porcine kidney when immunolabelled with the anti-APN primary antibody (a), but not when the primary antibody was replaced with rabbit IgG (b). In BHK-21 cells transfected with orthologues of APN plasmid, positive membranous and cytoplasmic immunolabeling was detected with the anti-APN rabbit monoclonal antibody (c-j), while no immunolabeling was observed in the no-plasmid control (k). Human (c), camel (d), sheep (e), greater horseshoe bat (f), greater mouse-eared bat (g), ferret (h), dog (i), cat (j). Images were taken at 200× (a,b) and 400x (c-k) magnification.

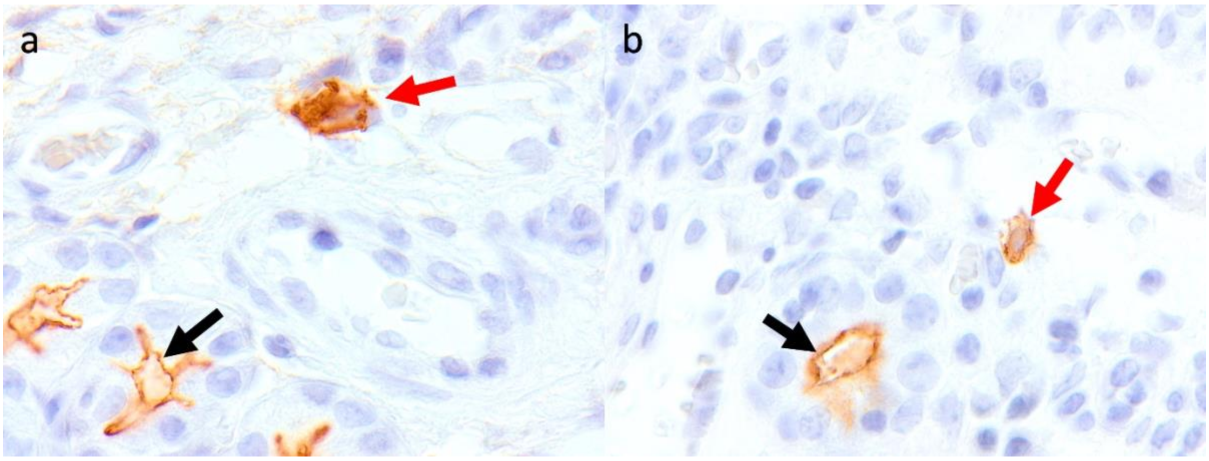

Figure S2. Immunohistochemical labelling of APN in the respiratory mucosa of the nasal turbinate in the domestic pig (*Sus scrofa domestica*; a) and wild boar (*Sus scrofa*; b). APN immunolabeling was observed on the apical surface of the submucosal glandular epithelium (black arrow) and within the cytoplasm of mononuclear round cells in the submucosa (red arrow). The size and morphology of these cells are suggestive of macrophages. Images were taken at 1000× magnification.

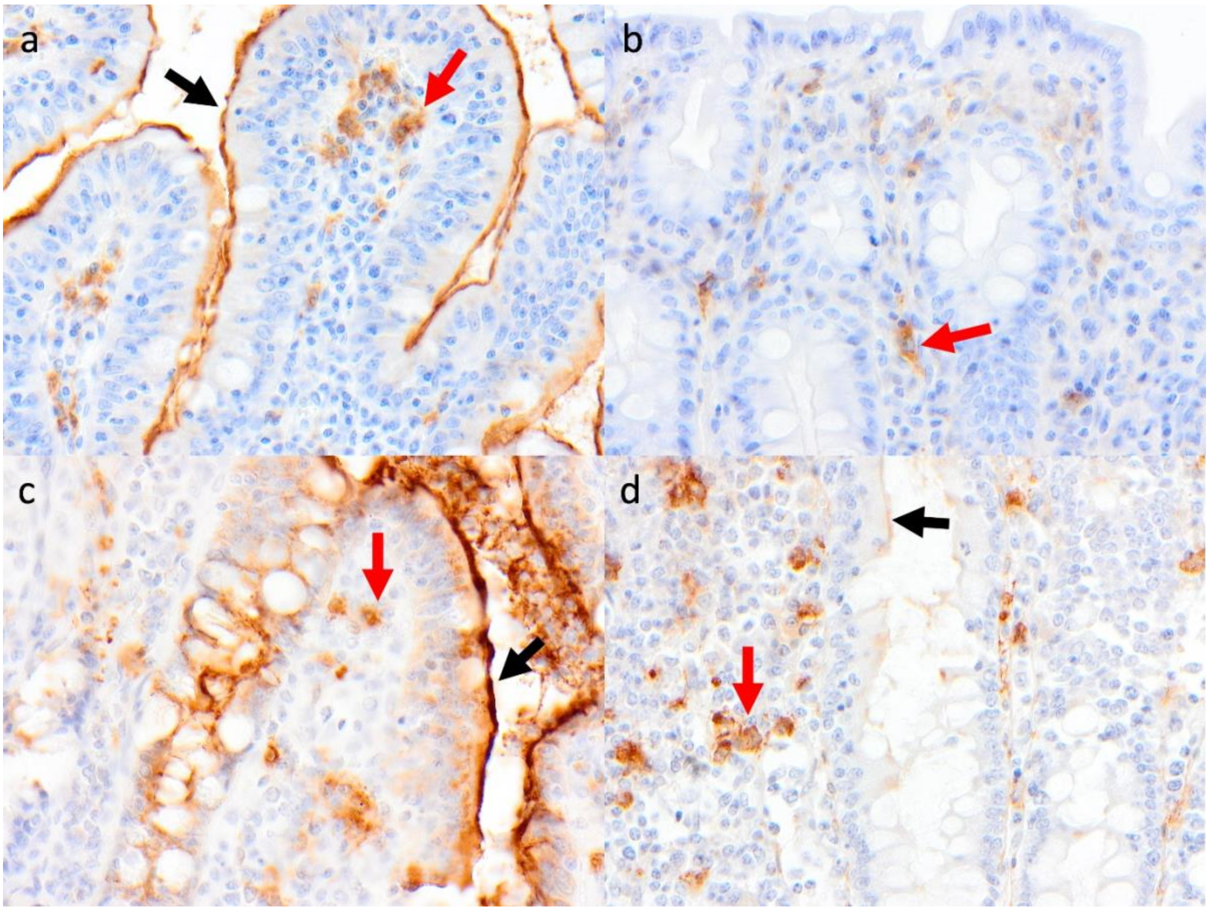

Figure S3. Immunohistochemical labelling of APN in the small intestine (a, c) and colon (b, d) of domestic pig (*Sus scrofa domestica*; a, b) and wild boar (*Sus scrofa*; c, d). Immunolabeling was observed on the apical aspect of enterocytes in the small intestine and colon (black arrow), along with scattered large, round immunopositive cells (red arrow) within the lamina propria. The size and morphology of these round cells are suggestive of macrophages. Images were taken at 400× magnification.
